# Words and Things

**Published:** 1918-02-09

**Authors:** 


### The Hospital

The Workers' Newspaper of Administrative Medicine and Institutional Life, Administration, National Insurance and Health.

No. 1653, Vol. LXIII. SATURDAY, FEBRUARY 9, 1918. PRICE ONE PENNY

### WORDS AND THINGS.

How difficult it is to foresee all the consequences of our acts, and how often our best meant acts have ill-consequences that come upon us as a surprise! We were all very indignant a few months ago that our soldiers who had lost their wits in the war should be treated as madmen, which they were, and we agitated to secure that if they were sent to asylums, the very best place for them, they should at any rate go as "private" patients and not as "pauper" patients. "Pauper" is an ill-sounding word, and we resented its application to men who had offered their lives to their country; but it is always weak and sometimes dangerous to allow ourselves to be governed by words. This is a lesson hard to learn, and that most people never learn. It has just been brought home by a stinging example to certain Poor-Law authorities. In order to clear them of the "stigma" of pauperism, soldiers sent to lunatic asylums were classified as "private" patients. But the petitioner upon whose petition an order for the detention of a "private" patient has been made, may at any time order the discharge of that patient; and if the petitioner makes an order for the discharge of a private patient, not the superintendent nor the committee of the asylum, nor all the King's horses nor all the Queen's men can legally prevent the discharge of that patient. Moreover there are cases in which the petitioner, who must in ordinary cases be a near relative of the patient, gains a pecuniary advantage, it may be a considerable pecuniary advantage, by the discharge of the patient. While the patient is in the asylum, his pension, or a considerable part of it, goes to pay for his support and for his treatment; but if he is taken out of the asylum the relatives appropriate the pension and may allot what part of it they please to the support of the patient, and are not bound to see that he gets any treatment at all. So it happens that some soldier-patients have been discharged by the order of a relative who gains, while the patient suffers, from the discharge. Such is the consequence of hasty and ill-considered action under the guiding influence of objection to a word.

It is curious that the very men who have had thus brought home to them the folly of legislating in order to get rid of their objection to a word, should attempt at the very same sitting to procure legislation to get rid of their objection to another word. This other word is "asylum," a harmless, gracious, and comforting word that was itself introduced to supersede the uncomfortable and objectionable word "madhouse." They, or their predecessors, were wretched until the madhouse was called an asylum, and now they are wretched until the asylum is called a mental hospital. No alteration of its name can make the asylum a mental hospital. If it were a mental hospital, it would exist in the mind of someone, and would have no material existence. It could be neither seen nor felt, nor could it receive patients, for no one can be received into another person's mind. What the sapient persons who speak of a mental hospital really mean is a hospital for mental diseases. We do not speak of a consumptive hospital, though there are many hospitals for consumption; nor do we speak of a venereal hospital, though there are hospitals for venereal diseases. Hospitals do not, in fact, suffer from consumption or from venereal diseases, and their founders recognise the fact; but the sapient alienist does not recognise that his asylum does not suffer from mental diseases, so he calls it a mental hospital. With what confidence can we entrust patients suffering from mental diseases to persons who know so little about the working of their own minds? And if the asylums were called hospitals for mental diseases, the term would still be a misnomer, for madness is, according to the most recent and authoritative view, not exclusively nor even mainly a mental disease, and there are many mental diseases that are far removed from madness, and that no one would dream of treating in an asylum. It would be difficult to find a better name for a madhouse than an asylum, and it would be equally difficult to find a worse name for it than a mental hospital. When will people learn that it is the thing, the madness, that is gruesome and objectionable, and not the place in which madmen reside? As long as madness is foolishly regarded with superstitious horror, so long will its name and the name of the place in which it is treated be regarded with horror equally superstitious and equally foolish. When people are sufficiently educated and sensible to regard madness as what it is—that is to say as a disease, comparable with other diseases—all this silly bother about its name and the name of the places in which it is treated will disappear. This is the object at which alienists should aim, and this object they entirely neglect. They have in their annual reports an easy and effectual medium for their propaganda, but they never take advantage of it. Is not the reason this: that they have not yet freed themselves from the superstition? It is the most obvious explanation, and it is difficult to find another that will fit the facts.

